# Lipids challenge ligands to control receptors

**DOI:** 10.7554/eLife.110543

**Published:** 2026-02-04

**Authors:** Adam JM Wollman

**Affiliations:** 1 Newcastle University Biosciences Institute, Newcastle University Newcastle upon Tyne United Kingdom

**Keywords:** lipids, receptors, tyrosine, tyrosinase, EGFR signalling, None

## Abstract

The behaviour of a receptor protein can be influenced by the presence of certain lipids in the membrane it is embedded in.

**Related research article** Srinivasan S, Lin X, Chen X, Regmi R, Zhang B, Schlau-Cohen GS. 2025. Active regulation of the epidermal growth factor receptor by the membrane bilayer. *eLife* **14**:RP108789. doi: 10.7554/eLife.108789.

The EGFR signalling pathway is central to many processes in cells (Sabbah et al., 2020). The pathway is activated when a ligand called EGF (epidermal growth factor) binds to a receptor protein called EGFR in the plasma membrane of a cell. Disruption of this signalling pathway can lead to diseases such as cancer and fibrosis.

For decades research into EGFR signalling has focussed on the ligand binding process and the subsequent protein-protein interactions that are triggered when the ligand binds to the receptor (Needham et al., 2016; Wollman et al., 2022). It was generally assumed that the plasma membrane was merely a passive scaffold in the binding process. Now, in eLife, Gabriela Schlau-Cohen and co-workers at MIT – including Shwetha Srinivasan as first author – report that the plasma membrane can be an active participant in the EGFR signalling pathway (Srinivasan et al., 2025). In particular, under certain circumstances, the plasma membrane can regulate – or even override – how the EGF receptor responds to its ligand.

The complexity of the plasma membrane has, in the past, made it difficult for researchers to dissect its contribution to EGFR signalling. To overcome this, Srinivasan et al. performed their experiments on nanodiscs – small circles made of lipid membrane that resemble the plasma membrane of a cell (Denisov and Sligar, 2016). These nanodiscs were notable for two reasons: first, a full-length EGF receptor protein was embedded in the nanodisc; second, Srinivasan et al. were able to vary the lipid content of the nanodiscs, and then observe how this influenced the response of the receptors.

The researchers used a technique called single-molecule Förster resonance energy transfer (FRET) to measure the receptor response (Srinivasan et al., 2022). This approach involved labelling two different regions of the receptor protein with two different coloured fluorescent dyes. When these dyes get closer together, the fluorescence they emit gets stronger, so recording the fluorescence with a sensitive microscope makes it possible to detect small changes in the conformation or shape of the receptor. This approach allowed Srinivasan et al. to probe how the intracellular region of the receptor, specifically the C-terminal tail and the kinase domain, moved relative to the membrane under different lipid conditions (Figure 1).

**Figure 1.**
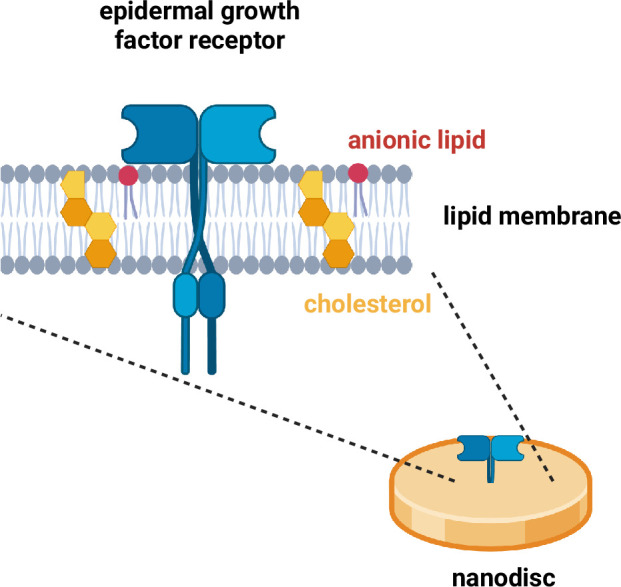
The EGFR signalling pathway is activated when a ligand binds to an EGF receptor protein that is embedded in the plasma membrane of a cell. Srinivasan et al. studied receptor proteins (blue) embedded in nanodiscs – small circles of lipid membrane (grey) that resemble the plasma membrane. Each receptor protein contains an N-terminal domain (top), a kinase domain, and a C-terminal tail (bottom). In particular, the researchers studied how the levels of two specific lipids in the membrane – anionic lipids (red) and cholesterol (yellow) – influenced how the receptor proteins responded to ligand binding. This schematic shows the receptor in an open, active conformation, with the C-terminal tail pointing away from the lipid membrane. Note: EGF receptor proteins pair up to form dimers. EGFR: epidermal growth factor receptor. Created with BioRender.com.

The investigation focused on two major classes of membrane lipids: anionic lipids and cholesterol, both of which are known to influence the activity of the receptor protein in complex ways. In healthy cells, the inner leaflet of the plasma membrane contains about 30% anionic lipids, such as POPS. The researchers studied nanodiscs with physiological levels of anionic lipids (15–30% POPS), and nanodiscs that did not contain any anionic lipids. Ligand binding activated the EGFR signalling pathway in both sets of experiments, but the former resulted in a larger conformational change in the intracellular domains of the receptor protein. This larger response suggests that anionic lipids enhance the responsiveness of the intracellular domains to the ligand.

Strikingly, at high concentrations of anionic lipids (60% POPS), which are characteristic of some cancer cells, the receptor entered a state of near-constant activity. The single-molecule FRET results showed the receptor was dominated by the “open” conformation – where the C-terminal tail is away from the lipid membrane – even in the absence of the ligand. This was further corroborated by high levels of ATP binding regardless of whether the ligand was present or not.

The mechanism underlying these effects is rooted in electrostatic interactions between the anionic lipids (which are negatively charged) and the receptor protein, which contains regions that are positively charged (the kinase domain and the basic residues in the region nearest the membrane) and regions that are negatively charged (the acidic residues in the C-terminal tail). The researchers propose a competition between the attractive forces (between the positively charged regions and the negatively charged anionic lipids) and the repulsive forces (between C-terminal tail and anionic lipids, both of which are negatively charged).

When the level of anionic lipids is high, the repulsive forces appear to dominate, driving the receptor into the open, active conformation: this means that EGFR signalling pathway can be activated without the ligand being present. This "constitutively active" conformation could be the biophysical origin of the enhanced signalling that fuels cancer cell growth.

In stark contrast, the presence of cholesterol – which changes the stiffness of cell membranes – effectively suppressed the response of the intracellular domains to ligand binding. It is known that cholesterol has an inhibitory effect on EGFR signalling (Ringerike et al., 2002), so this did not come as a surprise. Indeed, for all cholesterol-containing samples, the single-molecule FRET distributions were statistically the same in the presence and absence of the ligand. The mechanism responsible for the suppression of the response in these experiments appears to have its origins in membrane mechanics, rather than electrostatics. By comparing different lipids that influence membrane thickness or fluidity, Srinivasan et al. inferred that the decreased membrane fluidity caused by cholesterol is the likely culprit for the suppressed response.

These findings introduce a crucial new model for activation of the EGFR signalling pathway: in this model the lipid environment can essentially override ligand-driven control, thereby establishing a biophysical basis for both robust signalling in healthy cells and aberrant activity in pathology. The observation that the protein sequence likely responsible for this lipid dependence is conserved among the kinase domains of other receptor proteins suggests that active regulation by the plasma membrane may be a general feature of this entire class of proteins.

From a therapeutic point of view, these results suggest that researchers developing agents to target the EGFR signalling pathway may need to pay more attention to the plasma membrane around the receptor protein. Future studies can now investigate how the composition of this membrane contributes to the development and progression of diseases like cancer and neurodegenerative disorders.
